# A propos d'un cas de grossesse abdominale très prolongée

**DOI:** 10.11604/pamj.2013.16.26.2260

**Published:** 2013-09-25

**Authors:** Ignace Bwana Kangulu, Elie Kilolo Ngoy Umba, Deddy Kalonji Cibuabua, Clovis Mwamba Ilunga, Adellard Umba Ndolo, Michel Kabamba Nzaji, Prosper Kalenga Mwenze Kayamba

**Affiliations:** 1 Université de Kamina(R.D.Congo), faculté de médecine; 2 Institut Supérieur des techniques médicales de Kamina(R.D.Congo); 3 Université de Lubumbashi(R.D.Congo), faculté de médecine

**Keywords:** Grossesse abdominale, grossesse très prolongée, abdominal pregnancy, very prolonged pregnancy

## Abstract

Un cas de grossesse abdominale prolongée d'environ 18 mois avec mort fœtale, vécu à l'hôpital général de référence de Mulongo dans la province du Katanga, en République Démocratique du Congo, est rapporté dans ce papier. Ce cas clinique d’évolution étonnante permet de faire la revue de la littérature, de rappeler l'importance d'un bon suivi clinique et échographique de la grossesse et de s'interroger sur le niveau d’éducation de la femme ainsi que la qualité des soins prénataux en milieu rural congolais.

## Introduction

La grossesse abdominale, l'une des variétés des grossesses ectopiques, se définit comme étant l'implantation et le développement primaire ou secondaire de lœuf, en partie ou en totalité, dans la cavité abdominale. Les formes évolutives au-delà du 5è mois sont exceptionnelles dans les pays développés, mais fréquentes dans ceux à faible densité médicale [1–5]. Sa forme primitive est rare, de même que son évolution à terme émaillée d'une mortalité périnatale excessive. Souvent par défaut de moyens de réanimation suffisants et la précarité des infrastructures sanitaires dans les pays en développement, ce type de grossesse conduit aussi à une mortalité maternelle. C'est pourquoi, Correa l'a défini comme l′un des reflets du sous-développement [3, 4].

Dans nos milieux congolais (Congo-Kinshasa), connus peu équipés, la grossesse abdominale, favorisée par la recrudescence des séquelles des maladies sexuellement transmissibles, demeure encore une pathologie obstétricale relativement fréquente [6]. Dans les coins les plus reculés où l'accessibilité aux soins, la qualité de ces derniers et le taux d’éducation généralement faible sont à la base des évolutions étonnantes de plusieurs pathologies.

Dans cet article, nous rapportons un cas d'une grossesse abdominale très prolongée observé à l'hôpital général de référence de Mulongo (Hôpital des frères de Mulongo, dans le district sanitaire du Haut-Lomami, province du Katanga, en République Démocratique du Congo) au mois d'Avril 2012.

## Patient et observation

Il s'agit de la malade M, âgée de 22 ans, 3^e^ geste et 3^e^ pare, qui a vu ses dernières règles au mois d'Octobre 2010, admise à l'hôpital général de référence de Mulongo (Zone de santé de Mulongo au Katanga) en Avril 2012 pour douleurs abdominales généralisées, nausées surtout postprandiales, disparition des mouvements fœtaux actifs et notion de constipation intermittentes depuis plusieurs mois. La patiente souligne une durée exagérée de la grossesse malgré les différentes tentatives daccouchement provoqué, dans des postes de santé et à domicile déjà à partir du 11^e^ mois de gestation et la diminution progressive du volume abdominal. La grossesse na pas été suivie et on na enregistré aucun autre élément particulier dans les antécédents.

A l'examen physique, l’état général conservé, la tension artérielle de 90/50 mmHg, la température de 37.2° C et les conjonctives sont plus ou moins colorées. Labdomen est augmenté de volume, asymétrique avec le fœtus moulé à la paroi abdominale, la hauteur est de 28cm, la présentation transverse et les bruits cardiaques fœtaux à lauscultation sont absents. A l'examen gynécologique, le col est long, postérieur, ramolli et fermé. L'utérus est légèrement augmenté de volume mais dune manière non proportionnelle à l’âge de la grossesse.

**Figure 1:**
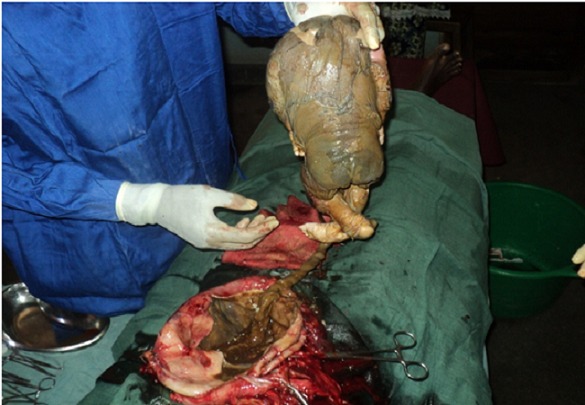
Nouveau-né macéré attaché à son cordon ombilical et son sac ovulaire vu au cours de la laparotomie exploratrice

L’échographie a montré un utérus augmenté de volume mais vide, un fœtus en présentation transverse dans la cavité péritonéale sans aucun signe de vie. La radiographie sans préparation de l'abdomen n'a pas été demandée en raison de son absence dans la structure. Une laparotomie médiane sous ombilico-suspubienne pratiquée sous anesthésie générale a permis de tomber directement sur le fœtus et ses annexes et nous avons constaté ce qui suit (Figure 1): un sac ovulaire dans la cavité abdominale, adhérant aux annexes utérines gauches, aux anses grêles, à l’épiploon et à la paroi pelvienne latérale droite, un utérus légèrement augmenté de volume, un fœtus fortement masséré avec petit placenta adhérant fortement à la paroi pelvienne latérale droite, une absence quasi-complète de liquide amniotique.

### Actes

A l'issue de toutes les observations ainsi dégagées, les actes suivants on été posés: Extraction d'un fœtus complètement masséré de sexe féminin pesant 2000g; clivage aux doigts du sac ovulaire suivi de la section aux ciseaux; tentative vaine d'extraction du placenta hypotrophié; vérification de l'hémostase; nettoyage de la cavité abdominale au sérum physiologique tiède; mise en place d'un drain tubulaire; fermeture de la paroi abdominale.

**Suites opératoires:** Infection pariétale traitée; la sortie de l'hôpital est intervenue au 17^e^ jour.

## Discussion

### Fréquence

La grossesse abdominale demeure une pathologie rare dans notre pratique quotidienne. Elle reste complexe quant-au diagnostic et la prise en charge en milieu rural, où le niveau d'instruction de la population est associé à un problème d'accessibilité aux soins de santé de qualité. Sa fréquence varie selon les régions. Elle est plus élevée dans les pays à faible densité médicale et le diagnostic y est également plus tardif [5, 7–12]. Sfar et al. à Tunis avaient rapporté le taux le plus bas: 1 sur 21439 naissances [13]. Chez nous, nous navons pas la fréquence exhaustive disponible de cette pathologie obstétricale.

### Caractéristiques maternelles

Ce cas en étude a concerné une jeune femme de 22 ans, paucipare, sans antécédents particuliers, vivant en milieu rural avec une grossesse non suivie. La grande particularité de cette gestation est son terme très dépassé sur une implantation ectopique. Le niveau d'instruction de la femme, la non fréquentation des bonnes consultations prénatales, le manque de diagnostic précoce de la pathologie sont autant des facteurs qui peuvent justifier cette évolution étonnante. Il y a lieu de s'interroger aussi sur la qualité du personnel soignant dans nos postes et centres de santé en milieu rural congolais.

La littérature montre que l’âge des patientes varie de 21 à 44 ans et la parité de zéro à trois enfants. Elle démontre que la fréquence de la grossesse abdominale dans nos milieux en développement semble être favorisée par 2 facteurs, l'infection génitale et l'insuffisance de suivi de la grossesse [9, 14–16].

### Diagnostic

Sur le plan clinique, plusieurs symptômes permettent d'orienter le diagnostic [17]:

- Les troubles digestifs: nausées, vomissements;
- Les douleurs abdominales et pelviennes concomitantes aux mouvements fœtaux si le fœtus est vivant avec ou sans métrorragies
- L'anémie avec altération de l’état général
- Un fœtus très superficiel souvent en position atypique transversale haute
- Parfois, une complication évolutive à type dhémorragie interne ou extériorisée, ou un syndrome toxi-infectieux
- Au toucher vaginal, le col est souvent fixé sous la symphyse pubienne, il est dur et long

La plupart de ces signes comme nous le notons en parcourant cet article, sont présents chez notre malade et ont plaidé en faveur du diagnostic.

La biologie peut montrer une anémie et une augmentation du taux de l'alpha-foeto-protéine [17]. Léchographie abdomino-pelvienne pratiquée nous a aidé à confirmer le diagnostic et nous a précisé la vitalité fœtale. La radiographie de labdomen sans préparation est indiquée en cas de syndrome occlusif ou dune position atypique du fœtus, mais lexamen clé demeure la cœlioscopie [18], inaccessible dans plusieurs structures hospitalières du pays comme dans celle de cette étude.

### Traitement et suites opératoires

La chirurgie est la seule sanction thérapeutique utilisée pour prendre en charge une grossesse abdominale. L'urgence opératoire est nuancée théoriquement selon la viabilité faetale [13].

En ce qui nous concerne, la viabilité fœtale était déjà exclue par la clinique et l’échographie et l'urgence était d'extraire le fœtus déjà mort en décomposition.

Nous devons noter que la grande particularité dans le déroulement de cette intervention chirurgicale est le sort réservé au placenta, lequel dépend de la localisation de son insertion. La décision dépend de l'inventaire des insertions du placenta. A cause du risque d'hémorragie incontrôlable, toutes tentatives d'extirpation sont formellement interdites si le placenta s'insère sur un organe noble ou sur un vaisseau [7, 8]. La résorption spontanée de ce placenta in situ est à contrôler par l’échographie et le dosage des hormones placentaires [10]. Cette surveillance est malheureusement difficile à réaliser dans nos conditions sous-médicalisées.

Dans notre cas, nous avons laissé le placenta «in-abdomino», vue son insertion et la crainte d'une hémorragie cataclysmale.

### Pronostic materno- fœtale

Concernant la grossesse abdominale, le pronostic fœtal est sombre avec une mortalité située entre 75% et 95% due à la vascularisation défectueuse du placenta (vieillissement précoce) à l'hypotrophie et aux malformations fœtales [19]. En plus, le grand dépassement de terme constaté dans ce présent cas peut s'ajouter à la liste des justifications de la mort fœtale.

Quant au pronostic maternel, il dépend du retard de diagnostic et de l'attitude prise vis-à-vis du placenta. Notre cas fait exception car la femme a évolué avec sa grossesse jusqu’à 18 mois, bien que le fœtus était déjà mort et une bonne évolution a été constatée en post-opératoire. Selon Hainaut, la mortalité maternelle varie de 0 à 18% [8].

## Conclusion

La grossesse abdominale, bien que rare, demeure une urgence obstétricale à laquelle on doit faire attention quant-au diagnostic et la prise en charge. La femme enceinte à l'obligation de fréquenter les bonnes consultations prénatales pour le suivi de la grossesse et le traitement éventuel des pathologies pouvant compromettre sa bonne évolution. Un personnel compétant dans des structures sanitaires rurales, capable de détecter les différentes anomalies de la grossesse et d'indiquer un traitement adéquat est aussi nécessaire.
